# Miscellaneous

**Published:** 1874-01-01

**Authors:** 


*MISCELLANEOUS.*

DR. MILTON PARKER.—At the meeting of the Chicago Medical Society, held on Monday evening, Dec. 15th, the following resolutions were passed relative to the death of Dr. Milton Parker:

WHEREAS, It has pleased Divine Providence to remove from our midst Dr. Milton Parker, after a long life of activity and usefulness,

*Resolved*, That in his death the medical profession has lost a competent and conscientious member, humanity an exemplar, and society an accomplished gentleman.

*Resolved*, That the Chicago Medical Society deeply deplores the bereavment of his family and friends, and extends to them its heartfelt sympathy.

*Resolved*, That a copy of these resolutions be furnished for publication to the medical journals and daily papers of the city, and that the family and friends be informed of the action of the Society.

---

J. D. TRITTON, a member of the class attending the Chicago Medical College, died on the 21st December, 1873. He was a member of the senior class, a good student, and a young man of much promise. He had been acting as one of the assistants in the Mercy Hospital, and enjoyed the full confidence and esteem of all his acquaintances. At a meeting of the students and members of the faculty the following resolutions were presented by a committee of the class and unanimously adopted:

*Resolved*, That in the death of J. D. Tritton society has lost a noble and worthy member, the profession a close student and promising active adjunct, and his friends a dear and true companion.

*Resolved*, That we deeply deplore the bereavement of his family, and extend to them our heartfelt sympathy.

*Resolved*, That a copy of these resolutions be furnished to the daily papres and THE MEDICAL EXAMINER for publication, and that his family be informed of our proceedings.

---

HAVE YOUR VOLUMES BOUND.—Any of our subscribers who wish can have their numbers of THE EXAMINER for 1873 bound in a handsome and durable style, in extra heavy cloth covers, for fifty cents per volume, by sending the numbers to our office, transportation both ways at expense of owner.

